# The Role of *Tbx20* in Cardiovascular Development and Function

**DOI:** 10.3389/fcell.2021.638542

**Published:** 2021-01-28

**Authors:** Yuwen Chen, Deyong Xiao, Lu Zhang, Chen-Leng Cai, Bai-Yan Li, Ying Liu

**Affiliations:** ^1^Department of Pharmacology, College of Pharmacy, Harbin Medical University, Harbin, China; ^2^Cardiovascular Developmental Biology Program, Herman B Wells Center for Pediatric Research, Indianapolis, IN, United States

**Keywords:** *Tbx20*, heart, development, function, congenital heart defects

## Abstract

*Tbx20* is a member of the Tbx1 subfamily of T-box-containing genes and is known to play a variety of fundamental roles in cardiovascular development and homeostasis as well as cardiac remodeling in response to pathophysiological stresses. Mutations in *TBX20* are widely associated with the complex spectrum of congenital heart defects (CHDs) in humans, which includes defects in chamber septation, chamber growth, and valvulogenesis. In addition, genetic variants of *TBX20* have been found to be associated with dilated cardiomyopathy and heart arrhythmia. This broad spectrum of cardiac morphogenetic and functional defects is likely due to its broad expression pattern in multiple cardiogenic cell lineages and its critical regulation of transcriptional networks during cardiac development. In this review, we summarize recent findings in our general understanding of the role of *Tbx20* in regulating several important aspects of cardiac development and homeostasis and heart function.

T-box (Tbx) family genes encode various transcription factors that are essential for embryonic development and organogenesis in the evolution of all metazoan, ranging from hydra to humans (Naiche et al., 2005). Cardiogenic lineage cells arise originally from the mesoderm, which is established during gastrulation (Moorman and Christoffels, 2003). *Brachyury* (also known as *T*) is considered the most ancient gene of the family and is critical to the formation and development of the posterior mesoderm (Herrmann et al., 1990); the loss of *Brachyury* impacts mesodermal specification and differentiation, resulting in a truncated tail in embryonic development (Papaioannou, 2001). The cardiogenic mesoderm between endodermal and mesodermal cells lumenizes and differentiates into endocardial cells (De Jong et al., 1990). Subsequently, the bilateral cardiogenic fields fuse in the midline to form the linear cardiac tube, which is followed by looping, septation, valvulogenesis, and chamber formation and maturation to become a functional heart (van den Berg and Moorman, 2009). The embryonic mesoderm of the mammalian embryo is built by a genetic network that involves master transcription factors and intracellular and intercellular signaling pathways. Previous studies have suggested that *T* is a direct downstream target of the Wnt3a signaling pathway, which provides a key balance between mesodermal and neuronal cell fates (Yamaguchi et al., 1999). In addition, another T-box-containing transcription factor, Eomesodermin (*Eomes*), plays an important role in early embryonic development, including mesodermal differentiation and migration as well as endoderm specification during gastrulation (Arnold et al., 2008). Similar to *Eomes, T* is also required for the formation, migration, and specification of nascent mesoderm cells (Morley et al., 2009). Along with the T-box family expanding throughout metazoan evolution (Papaioannou, 2014), family members became one of the most important master regulators in cardiovascular development, homeostasis, and function (Figure 1A). The importance of T-box family genes is further recognized by their profound contribution to inherited human disorders, including syndromic birth defects, congenital heart defects, dilated cardiomyopathy, and cardiac arrhythmogenesis (Packham and Brook, 2003; Papaioannou, 2014). The members of the *TBX1* subfamily (e.g., *TBX1, TBX18*, and *TBX20*) and *TBX2* subfamily (e.g., *TBX2, TBX3*, and *TBX5*) exhibit a comprehensive spatiotemporal expression pattern involved in all cardiogenic lineage cells, which are critical to cardiac development (Plageman and Yutzey, 2005; Stennard and Harvey, 2005; Just et al., 2016).

**Figure 1:**
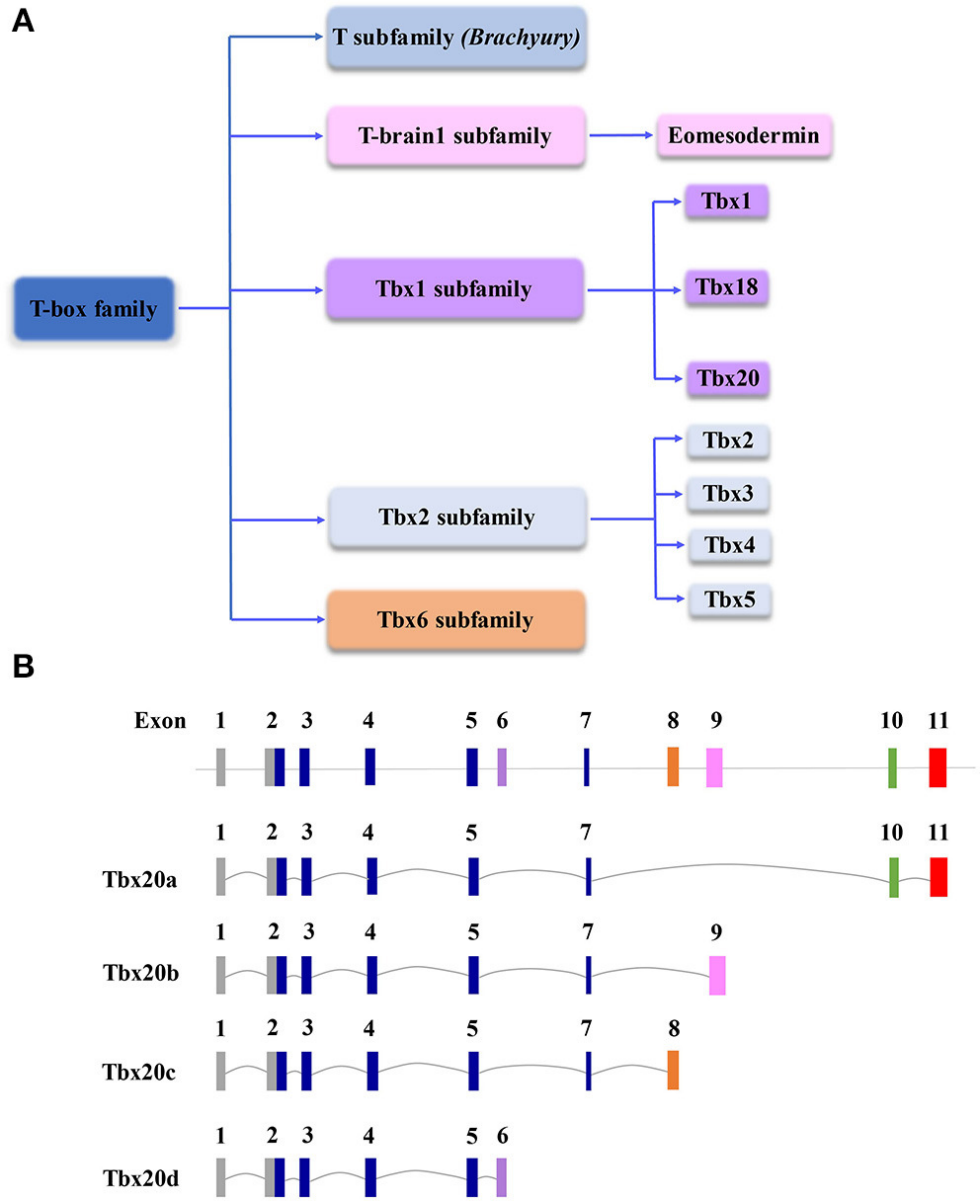
**(A)** Schematic diagram of the T-box gene family, which is important to cardiovascular development and function. **(B)** Schematic diagram of genomic structures of Tbx20 isoforms resulting from alternative splicing. Note, in the graph, the scales of introns and exons are not proportional to the exact sizes of each intron and exon.

*Tbx1* is expressed in the pharyngeal mesoderm and endoderm, outflow tract (OFT), and second heart field (SHF) (Xu et al., 2004). A set of common CHDs, including DiGeorge syndrome (also known as 22q11. 2 deletion syndrome), tetralogy of Fallot, double outlet right ventricle, and transposition of the great arteries, are attributed to *TBX1* mutations (Xu et al., 2004). *Tbx18* is expressed mainly in epicardial cells and myocardial sinus horns that descend from the embryonic venous pole. The expression pattern likely underlies the major developmental program, such as myocardialization of the caval veins and differentiation of the sinus node myocardium (Christoffels et al., 2006; Wiese et al., 2009; Greulich et al., 2016). The optimal cardiac vascular network is essential for efficient perfusion in the heart. *Tbx18*-deficient mice develop defective coronary conduit vessels largely due to altered proepicardial cell signaling and differentiation (Cai et al., 2008; Wu et al., 2013). In this review, we will focus on the role of *Tbx20* in cardiovascular development and function.

## *Tbx20* is an Evolutionally Conserved Master Regulator of Cardiac Development and Function

*Tbx20* is a member of the Tbx1 subfamily (Figure 1A). Originally identified in the E10.5 mouse heart cDNA library and named Tbx12, it comprises a 3.9 kb nucleotide sequence coding for a protein containing 446 amino acids (Carson et al., 2000; Kraus et al., 2001). There are at least four Tbx20 isoforms derived from alternative splicing, designated Tbx20a-d (Figure 1B) (Stennard et al., 2003). Tbx20a encodes a full-length protein containing a conserved T-box DNA-binding domain encoded by exons 2–5 and exon 7 flanked by N- and C-terminal domains, whereas 158 amino acids in C-terminal regions are equipped with strong transactivation and transrepression domains (Stennard et al., 2003; Hammer et al., 2008). Tbx20b and Tbx20c are alternatively spliced isoforms with alternative exons 9 and 8 at the 3′end, respectively. Both Tbx20b and Tbx20c contain T-box-binding domains but lack exon 10 (Stennard et al., 2003; DeBenedittis and Jiao, 2011). Tbx20d is the shortest isoform with a truncated T-box domain (Muller and Herrmann, 1997; Stennard et al., 2003). Unlike Tbx20d, all three other Tbx20 isoforms can bind to DNA, interact with Nkx2-5 and GATA4, and function in the cooperative or synergistic regulation of transcription (Stennard et al., 2003; DeBenedittis and Jiao, 2011). Tbx20a is only expressed in the heart, while Tbx20b is expressed more broadly (Takeuchi et al., 2005; DeBenedittis and Jiao, 2011). The overexpression of Tbx20a, but not Tbx20b, induces the mesodermal marker *Xbra*, endodermal marker *edd*, and cardiogenic marker *Nkx2-5*, suggesting that the Tbx20 isoforms provide important dynamic regulation of cardiovascular development (Stennard et al., 2003; DeBenedittis and Jiao, 2011).

*Tbx20* expression is found in almost all cardiogenic cell lineages throughout evolution from arthropod to vertebrate embryos (Ahn et al., 2000; Pocock et al., 2008). The Drosophila Tbx20 gene pair, neuromancer1 (*nmr1*, FlyBase:*H15*) and neuromancer2 (*nmr2*, FlyBase:*mid*), is expressed in early cardioblasts of the dorsal vessel (Griffin et al., 2000; Qian et al., 2005; Svendsen et al., 2009), a primitive heart-like organ in the fly. *H15/midline* is essential to the generation and maintenance of myofibrillar architecture and rhythmic contractile physiology (Qian et al., 2008). A series of studies demonstrated that Tbx20 in *Xenopus*, zebrafish, chicks, and mice functions in a very conserved fashion during heart development (Griffin et al., 2000; Iio et al., 2001). Tbx20 is one of the earliest markers in the *Xenopus* cardiogenic lineage. Tbx20 morpholino injection results in pericardial edema and reduced cardiac mass (Brown et al., 2003, 2005). Similarly, Tbx20 is important for driving cardioprogenitor formation and cardiomyocyte proliferation in zebrafish (Lu et al., 2017). The knockdown of Tbx20-homolog *hrT* in zebrafish leads to several cardiac developmental defects, including compromised cardiac looping and chamber formation (Szeto et al., 2002). Using explants from chick embryos, Tbx20 was shown to be involved in Bmp2 signaling in heart development (Plageman and Yutzey, 2004). These original findings indicate that Tbx20 has an indispensable role in cardiogenesis throughout evolution.

## *Tbx20* is Critical for Mammalian Cardiac Development and Function

*Tbx20* expression is found throughout mouse heart development in both cardiogenic heart fields (Kraus et al., 2001). *Tbx20* is expressed in the early cardiac progenitor region, endocardium and myocardium, endothelial cells of outflow track endocardial cushion and atrial ventricular cushion, the precursor structure of cardiac valves, and atrioventricular septum (Stennard et al., 2003). Temporally, *Tbx20* is found within the cardiogenic mesoderm as early as E7.5. As shown in *Tbx20* knockouts, *Tbx20* is required for heart looping and is essential for adequate proliferative activity in developing cardiomyocytes (Kraus et al., 2001; Cai et al., 2005; Stennard et al., 2005).

*Tbx20* knockouts die at ~E10.5 with severe hypoplastic ventricular walls, in conjunction with a defect in cardiac looping (Cai et al., 2005; Singh et al., 2005). Interestingly, *Tbx20* knockdown mice demonstrate a dose-dependent regulation of heart development; the knockdown mice exhibit OFT defects with a fused aorta and pulmonary arteries and hypoplastic right ventricles (Takeuchi et al., 2005). Consistent findings have been obtained in studies where gain-of-function Tbx20 mutations lead to a diverse array of cardiac defects, including abnormal ventricular walls, double outlet right ventricle (DORV), congenital atrial septal defects, patent foramen ovale (PFO), bicuspid aortic valve (BAV), and typical symptoms presented in familial tetralogy of Fallot (Posch et al., 2010; Zhang et al., 2011; Pan et al., 2015; Huang et al., 2017; Luyckx et al., 2019). In the adult heart, Tbx20 haploinsufficiency gives rise to left ventricular dilation, and systolic and diastolic dysfunction, which resembles dilated cardiomyopathy (DCM) in humans (Packham and Brook, 2003). Thus, *Tbx20* is not only important to heart development but also contributes to adult heart function, homeostasis, and physiological and pathophysiological adaptation (Stennard et al., 2005), which is further confirmed by severe dilated cardiomyopathy, arrhythmias, and heart failure in *Tbx20* conditional knockout adult cardiomyocytes (Shen et al., 2011). Collectively, these studies demonstrate the critical function of *Tbx20* in cardiac development and function.

## *Tbx20* Functions as a Key Transcriptional Modulator

**Figure 2:**
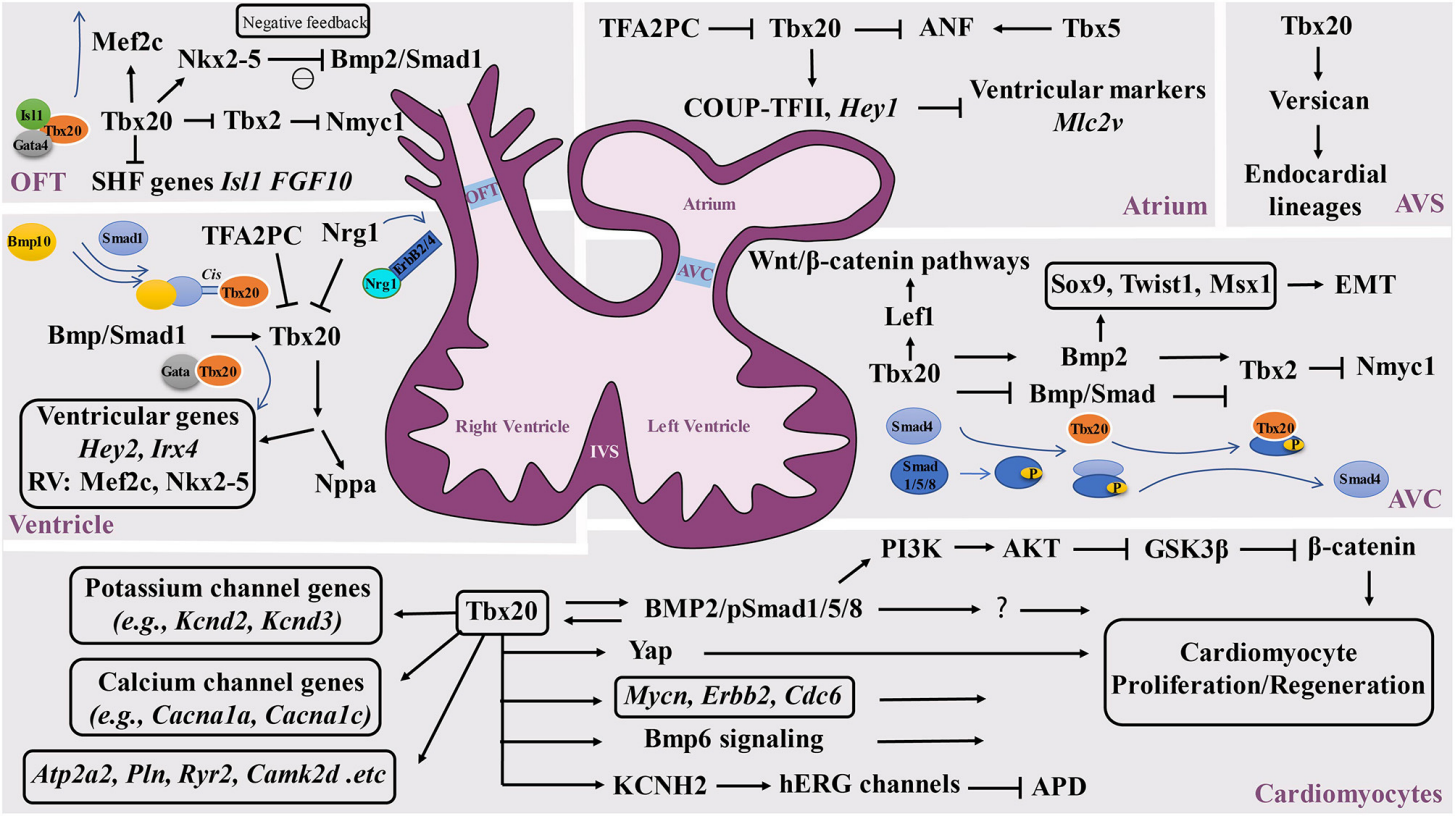
Schematic diagram of the Tbx20-mediated transcriptional network in cardiovascular development and function. *Tbx20* has a broad temporal and spatial expression profile and participates in a series of cardiogenic processes via its interaction with various transcriptional networks in developing and adult hearts. OFT, outflow track; AVC, atrioventricular cushion; AVS, atrioventricular septum; IVS, interventricular septum.

The broad expression pattern of *Tbx20* in the developing heart suggests that Tbx20 is involved in multiple cardiogenic processes by interacting with broad transcriptional networks in different regions of developing hearts (Figure 2). It has been shown that Tbx20 directly interacts with Nkx2-5, GATA4, and GATA5 to synergistically regulate cardiac gene expression in various cell types and coordinate cellular proliferation, differentiation, and chamber formation (Stennard et al., 2003; Brown et al., 2005). *Tbx20* knockout mouse embryos die at ~E10.5 with hypoplastic hearts, further confirming the importance of Tbx20 in cardiac development (Cai et al., 2005; Singh et al., 2005; Stennard et al., 2005). Its interaction with Nkx2.5 likely promotes cardiogenic progenitor cell proliferation and differentiation (Prall et al., 2007). Plageman and Yutzey showed that Tbx5 promotes natriuretic factor (ANF) expression, which is negatively regulated by Tbx20 (Plageman and Yutzey, 2004). Tbx20 has also been shown to function synergistically with Isl1 and Gata4 to activate *Mef2c* and *Nkx2-5*, which are required for normal formation of the right ventricle and outflow tract, providing a potentially unifying molecular mechanism for *Tbx20* as a transcriptional modulator in heart development (Takeuchi et al., 2005). Interestingly, the upregulation of *Isl1* is found in *Tbx20* mutant hearts (Cai et al., 2005). *Isl1* is a major determinant of cardiogenic progenitor cells. Chromatin immunoprecipitation (ChIP) analyses demonstrate that Tbx20 directly binds the conserved T-half sites within the *Isl1* promoter sequence, suggesting that Tbx20 is a negative regulator of *Isl1* expression (Cai et al., 2005).

In addition to negatively modulating Tbx5-mediated transcriptional function, Tbx20 also inhibits Tbx2-mediated transcriptional function and directly or indirectly represses *Tbx2* expression in the myocardium, providing a great stimulus to define the specification of the chamber and non-chamber myocardium, a lineage digression in the early stage of the heart underlying all subsequent morphogenesis (Stennard et al., 2005). In the developing outflow tract and atrioventricular canal, Tbx2 directly represses *Nmyc1*, a member of the Myc family of nuclear proto-oncogenes, resulting in relatively lower proliferative activity (Cai et al., 2005). Tbx20 can enhance proliferation efficiency by promoting *Nmyc1* expression (Cai et al., 2005). Additional analysis further demonstrated that Tbx20 can directly attenuate BMP/Smad signaling to suppress *Tbx2* expression in the chambers. Subsequently, *Tbx2* expression is confined to the developing atrioventricular canal region (Singh et al., 2009). It is important to highlight that *Tbx20* acts as an indirect repressor to restrict precocious *Tbx2* transcription by sequestering BMP-activated Smad1/5/8. The mutant isoforms of Tbx20 lacking DNA binding can bind to phosphorylated Smad1/5/8 and prevent the interaction between Smad1/5/8 andco-Smad4 (Stennard et al., 2003; Singh et al., 2009; Singh and Kispert, 2010). In addition, Mandel et al. also demonstrated that high-affinity Smad-binding sites are located in *Tbx20* cardiac regulatory elements. Blocking Smad-mediated signaling can specifically lead to the loss of Tbx20-mediated function (Mandel et al., 2010), further supporting the reciprocal regulation between the BMP/Smad pathway and Tbx20. Furthermore, Tbx20 is a direct regulator of COUP-TFII, which is necessary for the establishment of atrial identity; *Tbx20* conditional knockouts reduce the expression of COUP-TFII (Cai et al., 2003; Watanabe et al., 2012; Boogerd et al., 2018). Additionally, Tbx20 can directly regulate *Hey2, Irx4*, and genes that are essential to cardiomyocyte proliferation, such as *Mycn, Erbb2*, and *Cdc6* (Bersell et al., 2009; Boogerd et al., 2018; Ihara et al., 2020). In addition, Tbx20 can activate PI3K/AKT/GSK3β/β-catenin-dependent pathways and promote adult cardiomyocyte proliferation (Chakraborty et al., 2013). Another interesting finding is that Tbx20 overexpression in adult mouse hearts activates cardiomyocyte proliferation via Akt-, YAP-, and BMP-mediated signaling and represses inhibitory pathways via p21, Meis1, and Btg2 (Xiang et al., 2016). Apparently, Tbx20 overexpression in cardiomyocytes promotes myocardial repair or regeneration in adult hearts in response to myocardial injury, presenting a potential therapeutic strategy (Xiang et al., 2016; Fang et al., 2020). Furthermore, a study identified that myocardial Tbx20 induction enables the activation of the endocardium by promoting endocardial cell extension and proliferation at the injury site in adult zebrafish hearts, which likely occurs via the activation of endocardial bone morphogenetic protein 6 (Bmp6) signaling (Fang et al., 2020).

Another set of experiments aiming to determine the upstream events of *Tbx20* also revealed the key function of *Tbx20* in ventricular wall development. Neuregulin 1 (*Nrg1*), a member of the epidermal growth factor family, is known for its critical role in ventricular wall trabeculation (Lai et al., 2010; Del Monte-Nieto et al., 2018). Nrg1 is expressed in the endocardium but binds to its receptors ErbB2 and *ErbB4* in the myocardium to initiate trabeculation (Gassmann et al., 1995; Chang et al., 1997; Hertig et al., 1999; Bersell et al., 2009). *Nrg1* is found to suppress *Tbx20* expression in a dose-dependent manner, which highlights the potential mechanisms by which *Nrg1* can actively downregulate *Tbx20* during ventricular chamber maturation (Stennard et al., 2005). Similarly, Transcription factor AP-2 gamma (*Tfap2c*) was also found to be a transcriptional repressor of *Tbx20* expression. Decreased expression levels of *Tfap2c* can upregulate *Tbx20* expression (Hammer et al., 2008). More interestingly, several studies suggest that BMP-mediated signaling positively regulates *Tbx20* expression (Mandel et al., 2010; Zhang et al., 2011), suggesting that important positive and negative regulatory cascades regulate *Tbx20* expression during heart development.

*Tbx20* is known to be expressed in the endocardium. The endocardium is not only critical to ventricular wall development and maturation but also critical to valvulogenesis. The valves are derived from precursor structures known as endocardial cushions. A series of analyses revealed that *Tbx20* is required for early atrial ventricular cushion formation and endocardial endothelial-mesenchymal transformation (EMT). *Tbx20* is an important player in the modulation of extracellular matrix expression along with the promotion of cell proliferation in mesenchymal valve precursor structures derived from endocardial cushions (Shelton and Yutzey, 2007). An interesting finding is that Tbx20 initiates EMT action via the Bmp2-mediated regulation of *Sox9, Twist1*, and Msx1 expression in the developing atrial ventricular cushion (Ma et al., 2005; Cai et al., 2011). More importantly, Tbx20 can regulate *Lef1*, a key transcriptional mediator of the Wnt/β-catenin pathways, in the promotion of endocardial cushion maturation and valve elongation (Cai et al., 2013). In addition, Boogerd et al. demonstrated a direct role for Tbx20 in *Vcan* expression in endocardial lineages during septation (Boogerd et al., 2016), further suggesting the broad impact of Tbx20 in endocardial-mediated cardiogenic events.

## The Role of *Tbx20* in Cardiac Function

The propagation of electrical impulses that coordinate rhythmic and synchronized cardiac contractions to facilitate systemic circulation is regulated by the cardiac conduction system (CCS) in a spatiotemporally precise manner. The CCS is a complex set of specialized structures and cell types in the heart, which include the sinoatrial node (SAN), atrioventricular node (AVN), fast-conducting atrioventricular bundle (AVB), left and right bundle branches, and Purkinje fiber (PF) network. In addition, working cardiomyocytes also play a pivotal role in propagating electrical impulses throughout the myocardium. Congenital defects of the CCS and dysregulation of CCS homeostasis can lead to CCS dysfunction, causing life-threatening arrhythmias and increasing the risk of death in both children and adults (Wolf and Berul, 2006; Mangoni and Nargeot, 2008; Christoffels et al., 2010; Munshi, 2012; van Weerd and Christoffels, 2016). Genome-wide association studies (GWAS) in human patients with various arrhythmias have revealed a close association of abnormal electrocardiography (ECG) to many ion channels, gap junction proteins, muscle structural proteins and several critical transcription factors that function in the specification, differentiation and homeostatic maintenance of the CCS (van Weerd and Christoffels, 2016). These transcription factors include the T-box genes *TBX3, TBX5*, and *TBX20*. *Tbx3* and *Tbx5* are two known key transcriptional regulators of CCS development (van Weerd and Christoffels, 2016). Interestingly, although *Tbx20* was not initially associated with roles in CCS development, GWAS analysis revealed that single nucleotide polymorphisms (SNPs) within *TBX20* are linked to prolonged QRS duration with strong linkage disequilibrium (LD) (Sotoodehnia et al., 2010; Evans et al., 2016). These SNPs are intergenic, suggesting that these regions are involved in the transcriptional regulation of *TBX20*. These data suggest that *Tbx20* participates in the regulation of either CCS development/maintenance or myocardial conduction. It is likely that *Tbx20* coordinates and maintains the spatial and temporal control of the development and function of the cardiac conduction system via either parallel or single-gene regulatory pathways. Studies by Shen et al. demonstrated that mutant mice with conditional *Tbx20* ablation in adult cardiomyocytes have dilated hearts with a rapid loss of systolic function and slower conduction and severe arrhythmia (Shen et al., 2011; Sakabe et al., 2012). ChIP and enhancer analyses revealed a broad range of direct target genes of Tbx20 that contribute to regulating cardiac rhymical function (Sakabe et al., 2012). These downstream targets are largely linked to human inherited channelopathies (Priori and Napolitano, 2006; Lehnart et al., 2007; Roberts and Gollob, 2010; Shen et al., 2011). A recent study further demonstrated that Tbx20 selectively regulates the expression of *Kcnh2*, which encodes the channel Kv11.1 (hERG), a critical channel responsible for ventricular repolarizing currents. The human *TBX20* p.R311C mutation can lead to the loss of TBX20 transcriptional activity, which subsequently causes a lower expression level of hERG and inward rectifier current, leading to prolonged action potentials (Trudeau et al., 1995; Caballero et al., 2017). Moreover, Tbx20 is also found to regulate *Kcnd2, Kcnd3, Cacna1a, Cacna1c, Atp2a2, Pln, Ryr2*, and *Camk2d* (Shen et al., 2011), strongly supporting the critical role of Tbx20 in maintaining cardiac function.

In summary, this review summarized the recent findings related to our general understanding of the role of Tbx20 in regulating several important aspects of cardiac development, homeostasis, and heart function. These advances provide a basis for the early genetic diagnosis of associated CHDs, cardiomyopathies, and heart functional defects. However, the broad associations of Tbx20 with multiple biological events in multiple cardiogenic cell lineages during cardiac development make it difficult to map out all Tbx20-mediated upstream and downstream genetic networks intercellularly and/or intracellularly, especially the correlation of its genetic mutations found in complex CHD and cardiomyopathy patients with unique disease outcomes. Future analyses using hESC- and hiPSC-based analyses may help to confirm the findings from various animal models, thus addressing the key issues in disease progression in humans.

## Author Contributions

YC drafted the manuscript. DX and LZ helped with early revision. C-LC, B-YL, and YL finalized the manuscript. All authors contributed to the article and approved the submitted version.

## Conflict of Interest

The authors declare that the research was conducted in the absence of any commercial or financial relationships that could be construed as a potential conflict of interest.
